# Floating droplet electricity generator on water

**DOI:** 10.1093/nsr/nwaf318

**Published:** 2025-08-04

**Authors:** Wei Deng, Zihao Wang, Jingmin Wang, Tao Hu, Xiao Wang, Xuemei Li, Jun Yin, Wanlin Guo

**Affiliations:** State Key Laboratory of Mechanics and Control for Aerospace Structures, Key Laboratory for Intelligent Nano Materials and Devices of the Ministry of Education, Institute for Frontier Science, Nanjing University of Aeronautics and Astronautics, Nanjing 210016, China; State Key Laboratory of Mechanics and Control for Aerospace Structures, Key Laboratory for Intelligent Nano Materials and Devices of the Ministry of Education, Institute for Frontier Science, Nanjing University of Aeronautics and Astronautics, Nanjing 210016, China; Department of Materials Science, College of Materials Science and Engineering, Nanjing University of Aeronautics and Astronautics, Nanjing 210016, China; College of Aerospace Engineering, Nanjing University of Aeronautics and Astronautics, Nanjing 210016, China; College of Aerospace Engineering, Nanjing University of Aeronautics and Astronautics, Nanjing 210016, China; Department of Materials Science, College of Materials Science and Engineering, Nanjing University of Aeronautics and Astronautics, Nanjing 210016, China; State Key Laboratory of Mechanics and Control for Aerospace Structures, Key Laboratory for Intelligent Nano Materials and Devices of the Ministry of Education, Institute for Frontier Science, Nanjing University of Aeronautics and Astronautics, Nanjing 210016, China; College of Aerospace Engineering, Nanjing University of Aeronautics and Astronautics, Nanjing 210016, China; State Key Laboratory of Mechanics and Control for Aerospace Structures, Key Laboratory for Intelligent Nano Materials and Devices of the Ministry of Education, Institute for Frontier Science, Nanjing University of Aeronautics and Astronautics, Nanjing 210016, China; College of Aerospace Engineering, Nanjing University of Aeronautics and Astronautics, Nanjing 210016, China

**Keywords:** droplet electricity generation, water-integrated hydrovoltaic device, floating device, land-free applications

## Abstract

Hydrovoltaic technology holds great potential for energy harvesting from the natural water cycle. In this work, we present a novel water-integrated floating droplet electricity generator adopting a top electrode–dielectric–water architecture, where natural water acts as both the bottom electrode and substrate. The generator achieves high electrical output, comparable to the conventional counterpart using a metal bottom electrode and rigid substrate, while demonstrating 87% material weight reduction and 50% cost saving, as well as great durability in varying working environments. Its operational principle leverages the incompressibility and high surface tension of water to support the dielectric layer under droplet impinging and spreading, and the ion-rich composition of water enables exceptional high-frequency conductivity as the bottom electrode. The high surface tension of water also realizes unidirectional water transport for self-regulated water drainage. The advantages of water integration are further substantiated by the outstanding scalability, which is manifested by a sub-square-meter integrated device (∼0.3 m^2^). We anticipate this work will open up a new avenue of harnessing water-like natural materials to construct hydrovoltaic devices and advance land-free large-scale applications.

## Teaser

A floating droplet electricity generator leverages natural water, which is ion-rich, incompressible and of high surface tension, as the bottom electrode and substrate for device construction and advancing land-free large-scale applications.

## INTRODUCTION

Moving water droplets, such as raindrops, are widespread and carry a considerable amount of kinetic energy that could promise sustainable electricity generation. Harvesters based on conventional piezoelectric or electromagnetic effects generally rely on falling droplet-induced vibrations and suffer from low electrical output as well as vibration-caused material fatigue [1–4]. Leveraging contact electrification and droplet motion-induced formation receding of electrical double layers at the liquid–solid interface, the emerging hydrovoltaic technology offers a revolutionized avenue of electricity generation from water droplets [5–10]. In the past decade, droplet-based electricity generators (DEGs) have been receiving wide research efforts and are emerging as a promising approach to harvesting the kinetic energy of water droplets [11–28].

The prevailing DEG adopts an electrode–dielectric electrode–substrate structure. When a falling droplet impinges on the surface of the dielectric layer, the support from the rigid substrate allows the droplet to spread at high speed. Once the spreading droplet touches the top electrode, instantaneous charge transfer from the bottom electrode to the top electrode occurs, driven by electrostatic induction as the dielectric surface is generally negatively charged due to contact electrification [29–32], producing electrical output [12,13]. High peak output voltage at the level of hundreds of volts can be readily achieved from this type of double-electrode DEG, and strategies, such as augmenting surface charge [33,34], optimizing circuit capacitance [12], and engineering dielectric materials and electrode configuration [35–40], have been studied to improve the electrical output.

Determined by the electricity generation mechanism, that is, sequential contact electrification and electrostatic induction, the top electrode of a DEG is generally a thin metal wire, while the bottom electrode and substrate should be large enough to cover droplet spreading and support the dielectric layer. Therefore, innovating the current design of DEGs to reduce the use of the metal bottom electrode and rigid substrate may bring significant benefits in terms of materials cost, and facilitate large-scale applications. It has been reported that falling water droplets intentionally trapped in the reservoir between the dielectric layer and the substrate can work as the bottom electrode to construct a transparent DEG that is compatible with photovoltaic cells [26]. However, to our best knowledge, exploiting the multifunctionality of natural water for high-performance DEG construction has not been reported.

We herein propose a ‘nature-integrated’ design route, that is, leveraging natural materials, such as water, in an *in situ* manner to construct devices. Specifically, we develop a novel water-integrated floating DEG (W-DEG) that exploits the electrical and structural functions of water. When the dielectric layer (usually hydrophobic fluoropolymers) floats on the water surface, water naturally works as both the bottom electrode and substrate. The W-DEG has output performance comparable to the conventional DEG (C-DEG) with the metal bottom electrode and rigid substrate. We demonstrate that this is attributed to the exceptional electrical conductivity at high frequency and mechanical strength under high-speed impact of natural water, enabled by the incompressibility, high surface tension and ion-rich characteristics. Benefiting from using ‘free’ water as the building material, W-DEG features much lower materials cost and weight, as well as high potential for land-free applications. The W-DEG also shows great durability in varying working conditions. In addition, unidirectional water transport is achieved via leveraging water's high surface tension, to allow prompt water drainage in integrated devices. A sub-square-meter integrated device was fabricated easily, demonstrating the outstanding scalability arising from the water-integration strategy. We anticipate this nature-integrated design approach will advance the design of hydrovoltaic devices and land-free large-scale applications.

## RESULTS

**Figure 1.**
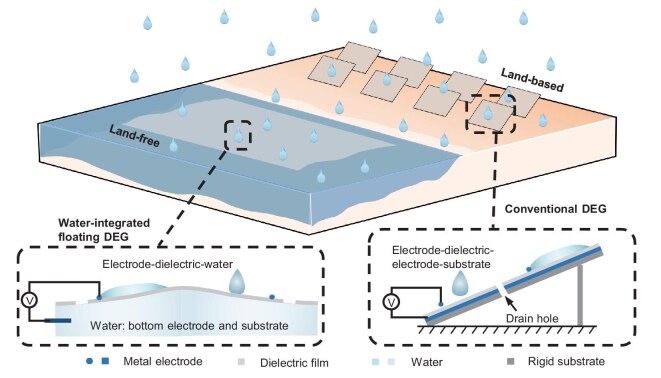
Schematic illustration of the C-DEG based on land and the W-DEG floating on water. The C-DEG has an electrode–dielectric electrode–substrate structure, generally supported on land. In comparison, the floating W-DEG has an electrode–dielectric–water structure, where water functions as both the bottom electrode and substrate, and features much lower materials weight and cost for potential large-scale land-free applications.

C-DEGs have a typical double-electrode structure, comprising metal top and bottom electrodes, a dielectric layer, and a rigid substrate, as well as adhesive layers between them when necessary (Fig. 1). In comparison, the novel floating W-DEG features a simplified electrode–dielectric–water structure via exploiting natural water as both the bottom electrode and substrate, which are usually metal tape and glass/plastic plates in C-DEGs. Note that a bottom electrode is essential as a single-top-electrode DEG produces orders of magnitude lower peak voltage output than the double-electrode DEG [17], and a rigid substrate is necessary to support the dielectric film and withstand milli-newton-level impacting forces exerted by the impinging droplets [41]. With the *in situ* use of ‘free’ natural water that is readily available, this nature-integrated design route transforms C-DEGs into W-DEGs of much lower weight and cost of materials, facilitating large-scale deployment. In addition, the floating W-DEG is inherently suitable for land-free applications that offer opportunities to utilize open water space and conserve land resources, which have not been explored before for DEGs.

To construct the W-DEG, a dielectric fluoropolymer film is floated on the water surface, with one end slightly lifted to make a tilt angle of ∼10°. A metal wire is fixed on top of the dielectric film as the top electrode and another one is inserted into water since water acts as the bottom electrode (Fig. 1). The C-DEG is constructed via a similar process except that copper tape is used as the bottom electrode, supported by an acrylic substrate. Tap water droplets successively fall onto the dielectric film and spread therein, during which the electrical output is recorded.

**Figure 2.**
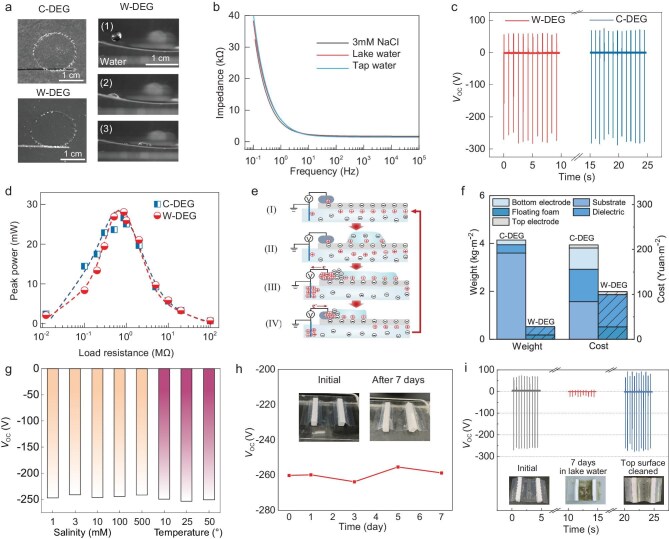
Output performance of the W-DEG and the C-DEG. (a) High-speed imaging of the droplets impacting on the dielectric film in the W-DEG (right, side view) and maximal droplet spreading in the W-DEG and C-DEG (left, top view). (b) Electrochemical impedance spectra of tap water, lake water and 3 mM NaCl solution. (c) Open-circuit voltage output of the W-DEG and C-DEG. Each droplet after spreading and recoiling was removed using a pipette to avoid interfering with the next droplet. (d) Peak power output of the W-DEG and C-DEG with varied load resistances. (e) Schematic illustration of the electricity generation mechanism of the W-DEG. (f) Comparison of the materials weight and cost between the W-DEG and C-DEG. (g) Voltage output of the W-DEG in NaCl solutions of varying salinities and temperatures. (h) Voltage output of the W-DEG after floating on collected lake water under simulated waving (50 r/min orbital shaking) in the lab for different days; insets show the digital photos of the W-DEG at the initial state and after 1 week. (i) Voltage output of the W-DEG before and after floating on outdoor lake water. The output was restored after cleaning the top surface of the dielectric layer. The insets show the digital photos of the device at the initial state, after floating for 1 week, and after cleaning the top surface.

We first investigate the mechanical and electrical properties of water to ascertain that water works competently as the bottom electrode and substrate. The dielectric layer can float on the water surface (Fig. S1). This is because the thin dielectric layer has a small areal density (0.035 g/cm^2^, Fig. 2f) and water has a high surface tension (72 mN/m) to support the hydrophobic dielectric layer, just like water striders with water-repellent legs can stand effortlessly on water [42]. When droplets impinge on the dielectric surface, the top-view digital images captured by the high-speed camera show that a droplet in the W-DEG has a maximal spreading area of 2.8 cm^2^, close to that of 2.9 cm^2^ in a C-DEG (Fig. 2a). In addition, the side-view images indicate that the dielectric layer undergoes negligible displacement under droplet impinging. These results signify that water underneath the dielectric layer behaves sufficiently ‘rigidly’ to withstand high-speed impact. This is because water is nearly incompressible and cannot flow away at a millisecond scale [43], thereby firmly supporting the dielectric layer to allow prompt droplet spreading for high electrical output. If the substrate is highly compressible, it will deform upon droplet impinging on the dielectric layer, resulting in the displacement of the latter. Consequently, the droplet experiences a smaller reaction force and reduced maximal spreading area [44], producing lower electrical output.

The electrical conductivities of commonly used natural water sources (e.g. tap water and lake water), and a 3 mM NaCl solution are assessed by electrochemical impedance spectroscopy (Fig. S2). As shown in Fig. 2b, the impedances of the three solutions are below 3 kΩ at frequencies higher than about 10 Hz, attributed to the resistive and capacitive characteristics of salt solutions. Note that the characteristic frequency of the output voltage pulse is approximately 20 kHz as the pulse rising time is about 0.05 ms (Fig. S3). Therefore, the impedances of the water electrode, even with different sizes and shapes (Fig. S4), are much smaller than the internal impedance of the W-DEG, which is at the million-ohm level (Fig. 2d), and natural water can replace the metal electrode with negligible effects on the output performance.

We then compare the electrical output performance of the W-DEG and C-DEG. High peak output voltage around 250 V is produced upon the droplets contacting the top electrode at maximal spreading in both the W-DEG and C-DEG (Fig. 2c). The current output and charge transfer amount of the W-DEG and C-DEG are also almost identical (Fig. S5). Similarly, the two DEGs exhibit nearly identical maximal peak power output at a load resistance of 0.87 MΩ (Fig. 2b). These results unambiguously demonstrate that the W-DEG has an output performance comparable to that of the C-DEG, because water works competently as the bottom electrode and substrate.

With the mechanical and electrical functions of water in W-DEGs clarified, the electricity generation process driven by contact electrification and electrostatic induction is proposed, as illustrated in Fig. 2e. In state I, the top surface of the dielectric layer is negatively charged due to contact electrification at the dielectric–water interface [28–31] and positive ions in the bottom water electrode are electrostatically attracted to the bottom surface of the dielectric layer. In state II, a water droplet impinges and subsequently spreads on the top surface of the dielectric layer. Positive ions in the droplet are electrostatically attracted to the top surface of the dielectric layer. In state III, the spreading droplet contacts the top electrode. At this moment, a closed-loop circuit forms and negative ions in the droplet quickly migrate to the top electrode–water interface while positive ions stay at the dielectric–water interface. As a result, electrons transfer instantaneously to the electrical lead inserted in the bottom water through the external circuit, producing a pulse output. Meanwhile, the lead becomes negatively charged and attracts positive ions. When the droplet recoils, in state IV, electrons flow back to the top electrode and positive ions in the bottom water migrate back to the bottom surface of the dielectric layer. After the droplet leaves the dielectric surface, the W-DEG returns to state I. This process is similar to that in the C-DEG [12], except that the electrons migrate in the bottom metal electrode in the C-DEG (Fig. S6) while ions migrate in water in the W-DEG.

Besides the electrical output performance, the materials cost and weight, essential for practical applications, between C-DEGs and W-DEGs are also compared (Fig. 2f). For C-DEGs, the materials cost mainly comes from the dielectric film, bottom metal electrode and substrate, and the total cost is about 210 Yuan·m^−2^ (calculated from the retail prices of the materials), of which the latter two materials account for about 63%. In comparison, without the need for the bottom metal electrode and substrate, the cost of W-DEGs is about 106 Yuan·m^−2^, only half of that of C-DEGs. Regarding the weight, the area density of C-DEGs is about 4.14 kg·m^−2^ and the rigid substrate accounts for about 90% of it. In contrast, W-DEGs weigh about 0.5 kg·m^−2^, only 13% of that C-DEGs, which can greatly facilitate the transportation and deployment, further reducing application cost.

The output performance and durability of the W-DEG under varying environmental conditions are also assessed. The voltage output of the W-DEG does not vary significantly under different temperatures (10°C, 25°C and 50°C) and water salinities (1, 3, 10, 100 and 500 mM NaCl), as shown in Fig. 2g. The W-DEG experiences negligible performance degradation after floating on water of high salinity (500 mM NaCl) for 1 week and this is expected since the dielectric material, fluorinated ethylene propylene (FEP), is chemically inert (Fig. S7). Similarly, biofouling on the bottom surface does not appreciably affect the output voltage, as evidenced by the tests of the W-DEGs floating on collected lake water in the lab and outdoor lake water (Fig. 2h and i), where clear microorganism growth in the water and on the bottom surface of the dielectric layer are observed (Fig. S8). This is reasonable because microorganisms are water-rich and will not impede ion migration under electrostatic induction. While biofouling on the bottom surface of the dielectric layer does not affect the output, it should be noted that lake water splashing onto the top surface during raining leaves contaminants therein, harming the output performance of the W-DEG. These contaminants can be easily removed, restoring the output voltage. In outdoor applications, adding protective walls at the edges may help prevention of water-splashing-caused contamination therein and maintain the electrical performance. The output performance of the W-DEG after floating on simulated wavy water surface (50 r/min orbital shaking) and outdoor lake water (Fig. 2h and i) also indicates high structural integrity of the W-DEG.

It is worth emphasizing that the optimal efficiency of DEGs operating in the impinging–spreading mode is achieved by matching the timescales of the external circuit and the droplet-spreading process [15]. In simple terms, to achieve high output, droplets need to contact the top electrode at maximum spreading. However, this remains challenging for real raindrops with varying sizes, falling speeds and impacting positions. The dielectric film uniformity and integrity under wave disturbances of W-DEG devices are also essential in large-scale and practical applications. Therefore, further research is needed to address the challenges posed by the diverse real raindrops, and to improve the uniformity and integrity of W-DEG devices to ensure practical implementation.

**Figure 3.**
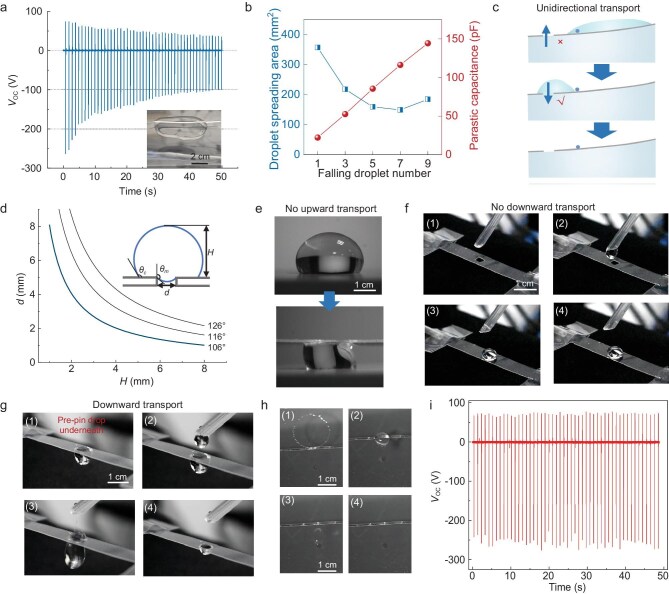
Surface tension-enabled unidirectional water drainage in the W-DEG. (a) Voltage output decrease caused by water accumulation on the top surface of the dielectric film as no drain hole is added. (b) Water accumulation induced increase of the parasitic capacitance between the top and bottom electrodes, and decrease of the droplet spreading area. (c) Schematic diagram shows desired unidirectional water transport through the drain hole. (d) Dependence of the critical hole size on the droplet height with different meniscus angles; inset illustrates the droplet residing on top of the hole. (e and f) Digital photos show that the droplet does not transport upward or downward through the hole under a certain pressure when the other side is air. (g) Digital photos show that the droplet spontaneously transports downward through the hole when pre-pinning water is beneath the hole. (h) Digital photos show that the falling droplet after spreading and recoiling promptly drains downward through the hole in the W-DEG. (i) Stable voltage output of the W-DEG with a drain hole.

In potential large-scale applications, prompt water drainage is crucial in DEG integration to avoid water accumulation or downstream water flow interfering with other droplets [36]. As shown in Fig. 3a, without a drain hole, the peak output voltage of the W-DEG decreases gradually as droplets fall on the dielectric surface and accumulate therein. We reveal that there are two main causes (Fig. 3b). On the one hand, accumulated water electrically bridges the spreading droplets with the top electrode before maximal spreading, which decreases the water–solid interface area and charge transfer amount induced by electrostatic induction. On the other hand, accumulated water enlarges the overlapping area between the top and bottom electrodes and the resultant parasitic capacitance, decreasing the voltage output, as can be inferred from the equivalent circuit [12]. As such, drain holes are indispensable to direct water flow to promptly leave the top surface of the dielectric film [36].

The water-integrated feature of W-DEGs necessitates the rational design of the drain holes to allow water transport downward while preventing transport upward, that is, unidirectional water transport (Fig. 3c). This can be achieved, leveraging the hydrophobicity of dielectric film (e.g. fluoropolymers) and high surface tension of water, through optimizing the hole size. By balancing the forces due to gravity and surface tension, the critical pore size is derived by Choi and Lang [45] as


(1) \begin{eqnarray*}
d = \frac{{ - 4\gamma {\rm cos}\, {\theta }_m}}{{\rho gH}},
\end{eqnarray*}

where $\gamma $, ${\theta }_m$, $\rho $, *g* and *H* are the surface tension of water, meniscus angle, water density, gravitational acceleration and droplet height, respectively (Fig. 3d). Assuming the meniscus angle is close to the apparent water contact angle on the hydrophobic dielectric film (${\theta }_c\ $∼ 106° ± 2°, Fig. S9), the dependence of the critical hole size on the droplet height is obtained. The droplet would be inhibited to flow through the hole when the hole size (*d*) and droplet height (*H*) reside beneath the *d–H* curve. Increasing the pore size or droplet size (i.e. hydrostatic pressure) leads to the deformation of the meniscus in a metastable state, and eventually the droplet penetrates through the pore. Note that the meniscus angle can be better approximated by the advancing contact angle that is larger than the apparent contact angle, which would shift the *d–H* curve upward. Therefore, the apparent contact angle gives the lower boundary of the critical hole size, which is used to guide the drain hole design.

Based on the analysis above, we choose the 3-mm rectangle hole as the drain hole for the W-DEG and we experimentally verified that when the hole size is larger than 3 mm, water starts to flow upward through the hole under hydraulic pressure (Fig. S10). When pressing a water droplet with the dielectric film against the drain hole, the droplet deforms but does not transport upward through the hole (Fig. 3e), sustained by water surface tension. Similarly, the droplet residing on top of the dielectric film does not transport downward through the hole (Fig. 3f). Therefore, when the dielectric film floats on the water surface, no water will penetrate the drain hole. When a droplet is pinned beneath the dielectric film, mimicking the film floating on water, the top droplet will make contact with the bottom one through the hole. As this eliminates the water–air interface and the corresponding upward force due to surface tension, the top droplet quickly transports downward through the hole, merges with the bottom droplet, and detaches from the dielectric film driven by gravity (Fig. 3g). In this way, unidirectional water transport is achieved for self-regulated water drainage. When the W-DEG is equipped with the designed drain hole, the falling droplets, after spreading and recoiling, can quickly leave the top surface of the dielectric film (Fig. 3h). Consequently, the voltage output of the W-DEG with a drain hole remains stable for consecutive falling droplets (Fig. 3i).

The water-integrated feature endows the W-DEG with exceptional scalability as the need for metal bottom electrodes and rigid substrates is obviated. In comparison, for C-DEGs using a metal bottom electrode and rigid substrate, one promising integration strategy is adopting commercial printed circuit board technology to facilitate the scalable fabrication of DEG arrays, and such a fabrication route reduces the number of output interfacing nodes and eliminates the complicated wire bridging [36,46,47].

**Figure 4.**
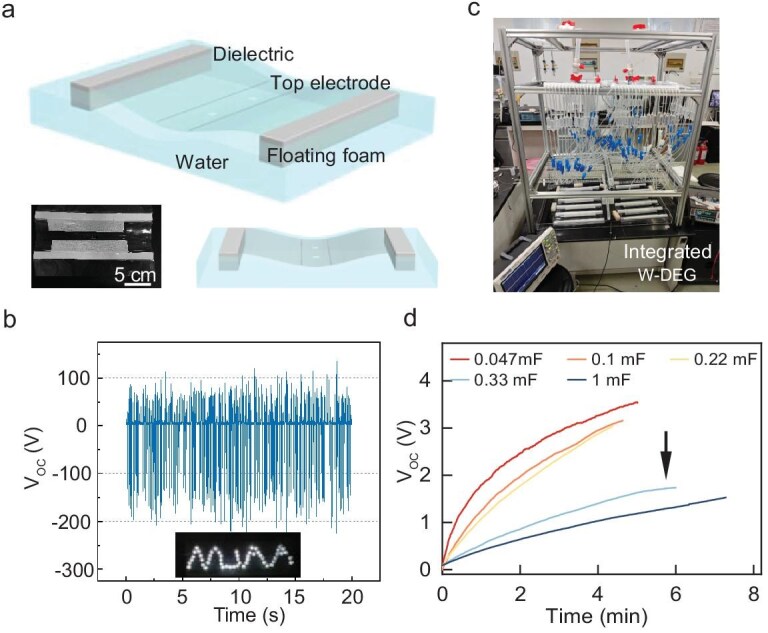
Output performance of the integrated W-DEG. (a) Schematics and digital photo of a W-DEG unit device. (b) Voltage output of the W-DEG unit under droplets from 12 droppers; inset shows that commercial LEDs were illuminated by the unit device. (c) Digital photos of the integrated W-DEG device composed of 10 units and the testing setup. (d) Voltage profiles of the capacitors charged by the integrated W-DEG.

To accommodate multiple falling droplets, a trough-shaped W-DEG unit is designed composed of two floating blocks, a dielectric film and a top wire electrode. Drain holes are located at the middle of the trough to allow water droplets to flow down after impinging (Fig. 4a). The unit device is tested under falling droplets from 12 droppers and the dropping frequency is about 1.5 Hz. Multiple output voltage pulses are observed (Fig. 4b), indicating that each droplet produces a pulse independently. The peak voltage output is about 200 V, slightly lower than with only one dropper (Fig. 2c), possibly caused by droplets splashing during impinging and the output of the DEG being alternative current. The unit device could almost unremittingly illuminate 50 commercial LEDs (Supplementary Video 1), demonstrating the feasibility of powering electronics.

To further substantiate the scalability, a 0.3 m^2^ integrated device, consisting of 10 W-DEG units (Fig. S11), was fabricated with ease, which is much larger than previously reported integrated DEGs [13,36]. A homemade droplet dispensing platform comprising 120 droppers was used for the testing of the integrated device. To improve energy harvesting from the pulse-like output of the DEG, a power management circuit composed of full-wave rectifiers and inductors is adapted and the energy is stored in capacitors (Fig. S12). The integrated W-DEG quickly charges capacitors, as high as 0.22 mF, to 3 V within a few minutes. For the 0.33 mF capacitor, 1.8 V is achieved in 6 mins. Even for the 1 mF capacitor, a voltage over 1.5 V is achieved. The exhibited high electrical output of the integrated DEG promises applications, such as powering wireless sensors for water-quality monitoring.

## CONCLUSION

In summary, by exploiting the electrical and structural functions of water, we develop a novel water-integrated floating DEG, wherein water naturally works as both the bottom electrode and substrate. The W-DEG exhibits hundred-volt-level peak voltage output, comparable to the conventional DEG with a metal bottom electrode and rigid substrate, but has much lower materials weight and cost. It is revealed that the exceptional electrical conductivity at high frequency and mechanical strength under high-speed impact of natural water contribute to the high output performance of the W-DEG. The W-DEG also shows great durability in varying working environments. In addition, unidirectional water transport is achieved, leveraging water's high surface tension, to allow prompt water drainage. The W-DEG shows great scalability, demonstrated by a sub-square-meter integrated device. Benefiting from using ‘free’ and omnipresent water as the building material, without compromising output performance, we anticipate this nature-integrated design approach will advance the design of hydrovoltaic devices and land-free large-scale applications.

## MATERIALS AND METHODS

### Device fabrication

To fabricate the conventional double-electrode DEG (C-DEG), a piece of copper tape, as the bottom electrode, was fixed onto a 3-mm thick acrylic plate using adhesives. A 0.2-mm thick FEP film was then attached to the glue side (top side) of the copper tape. A metal wire (diameter: 0.05 mm) was fixed on top of the FEP film to work as the top electrode. The C-DEG was inclined at an angle of about 10° relative to the horizontal plane during test. The W-DEG was fabricated without using copper tape and acrylic plate; instead, the FEP film floated on water surface. One side of the FEP film was slightly lifted by a foam to make an incline angle of about 10°. Drain holes were added onto the FEP film close to the top electrode. Since water worked as the bottom electrode, a metal wire was inserted into water for output measurement. To fabricate the W-DEG unit that could accommodate multiple falling droplets, two floating foams were used to lift the two ends of the FEP film to maintain the tilt angle of about 10°. During operation, plastic blocks were put beside the device to keep it from drifting. The integrated W-DEG was fabricated by assembling 10 unit devices. Slices of air-laid paper were attached to the bottom surface on the lifted region of the FEP film to wick water and ensure good contact between water and the FEP film.

### Characterization and measurements

The droplet-dispensing platform comprising 120 droppers was built using commercial infusion apparatus. Tap water with a salinity of ∼0.02% was used for DEG testing. The output voltage of the DEG was measured by an oscilloscope (Siglent SDS2352X Plus with a 1:100 probe) and the current was recorded with an additional noise current preamplifier (Stanford Research System, SR570). Charge transfer was obtained on an electrometer (KEITHLEY 6517B) and the voltage of capacitors charged by the integrated W-DEG was acquired using a Keithley digital multimeter (DMM 6500). Droplet spreading dynamics were recorded with a high-speed camera (AMETEK, Phantom VEO 1310 L) and the electrochemical impedance spectra of tap water, lake water and 3 mM NaCl solution were obtained using an electrochemical workstation (AMETEK, PARSTAT 3000A-DX). To assess the structural integrity, as well as the effects of biofouling and possible corrosion on the output performance, the W-DEGs were tested after floating on collected lake water under simulated waves (50 r/min orbital shaking) in the lab, in a 500 mM NaCl solution and in outdoor lake water (Donghua lake, Nanjing, China).

## Supplementary Material

nwaf318_Supplemental_Files

## References

[1] Ma Z, Ai J, Shi Y et al. A superhydrophobic droplet-based magnetoelectric hybrid system to generate electricity and collect water simultaneously. Adv Mater 2020; 32: 2006839. 10.1002/adma.202006839 33179284

[2] Zhang X, Wang Q, Zou R et al. 3D-printed superhydrophobic and magnetic device that can self-powered sense a tiny droplet impact. Engineering 2022; 15: 196–205. 10.1016/j.eng.2022.04.009

[3] Bao B, Wang Q. A rain energy harvester using a self-release tank. Mech Syst Signal Process 2021; 147: 107099. 10.1016/j.ymssp.2020.107099

[4] Ilyas MA, Swingler J. Piezoelectric energy harvesting from raindrop impacts. Energy 2015; 90: 796–806. 10.1016/j.energy.2015.07.114

[5] Zhang Z, Li X, Yin J et al. Emerging hydrovoltaic technology. Nat Nanotechnol 2018; 13: 1109–19. 10.1038/s41565-018-0228-6 30523296

[6] Wang X, Lin F, Wang X et al. Hydrovoltaic technology: from mechanism to applications. Chem Soc Rev 2022; 51: 4902–27. 10.1039/D1CS00778E 35638386

[7] Hu T, Zhang K, Deng W et al. Hydrovoltaic effects from mechanical–electric coupling at the water–solid interface. ACS Nano 2024; 18: 23912–40. 10.1021/acsnano.4c07900 39168863

[8] Li L, Wang X, Deng W et al. Hydrovoltaic energy from water droplets: device configurations, mechanisms, and applications. Droplet 2023; 2: e77. 10.1002/dro2.77

[9] Yin J, Li X, Yu J et al. Generating electricity by moving a droplet of ionic liquid along graphene. Nat Nanotechnol 2014; 9: 378–83. 10.1038/nnano.2014.56 24705513

[10] Yin J, Zhang Z, Li X et al. Waving potential in graphene. Nat Commun 2014; 5: 3582. 10.1038/ncomms4582 24800734

[11] Xu W, Zhou X, Hao C et al. SLIPS-TENG: robust triboelectric nanogenerator with optical and charge transparency using a slippery interface. Natl Sci Rev 2019; 6: 540–50. 10.1093/nsr/nwz025 34691903 PMC8291521

[12] Li L, Li X, Deng W et al. Sparking potential over 1200 V by a falling water droplet. Sci Adv 2023; 9: eadi2993. 10.1126/sciadv.adi2993 37967189 PMC10651119

[13] Xu W, Zheng H, Liu Y et al. A droplet-based electricity generator with high instantaneous power density. Nature 2020; 578: 392–6. 10.1038/s41586-020-1985-6 32025037

[14] Wang X, Fang S, Tan J et al. Dynamics for droplet-based electricity generators. Nano Energy 2021; 80: 105558. 10.1016/j.nanoen.2020.105558

[15] Wu H, Mendel N, van den Ende D et al. Energy harvesting from drops impacting onto charged surfaces. Phys Rev Lett 2020; 125: 078301. 10.1103/PhysRevLett.125.078301 32857530

[16] Zhang H, Tian B, Jiang X et al. Dynamical mechanism for reaching ultrahigh voltages from a falling droplet. Adv Funct Mater 2024; 34: 2315912. 10.1002/adfm.202315912

[17] Li X, Ning X, Li L et al. Performance and power management of droplets-based electricity generators. Nano Energy 2022; 92: 106705. 10.1016/j.nanoen.2021.106705

[18] Li L, Li X, Yu X et al. Boosting the output of bottom-electrode droplets energy harvester by a branched electrode. Nano Energy 2022; 95: 107024. 10.1016/j.nanoen.2022.107024

[19] Wang X, Hu T, Wang X et al. Moving water droplets induced electricity on an electret surface with a charge gradient. Nano Energy 2023; 117: 108918. 10.1016/j.nanoen.2023.108918

[20] Li Y, Zhang Q, Cao Y et al. A constant-current generator via water droplets driving Schottky diodes without a rectifying circuit. Energy Environ Sci 2023; 16: 4620–9. 10.1039/D3EE02280C

[21] Zheng H, Wu H, Yi Z et al. Remote-controlled droplet chains-based electricity generators. Adv Energy Mater 2023; 13: 2203825. 10.1002/aenm.202203825

[22] Zhang N, Gu H, Zheng H et al. Boosting the output performance of volume effect electricity generator (VEEG) with water column. Nano Energy 2020; 73: 104748. 10.1016/j.nanoen.2020.104748

[23] Li X, Feng G, Chen Y et al. Hybrid hydrovoltaic electricity generation driven by water evaporation. Nano Res Energy 2024; 3: e9120110. 10.26599/NRE.2024.9120110

[24] Yang S, Su Y, Xu Y et al. Mechanism of electric power generation from ionic droplet motion on polymer supported graphene. J Am Chem Soc 2018; 140: 13746–52. 10.1021/jacs.8b07778 30257558

[25] Park J, Song S, Yang Y et al. Identification of droplet-flow-induced electric energy on electrolyte–insulator–semiconductor structure. J Am Chem Soc 2017; 139: 10968–71. 10.1021/jacs.7b05030 28753025

[26] Jang S, Shah SA, Lee J et al. Beyond metallic electrode: spontaneous formation of fluidic electrodes from operational liquid in highly functional droplet-based electricity generator. Adv Mater 2024; 36: 2403090. 10.1002/adma.202403090 38695508

[27] Hu Y, Yang W, Ma Y et al. Solid-liquid interface charge transfer for generation of H_2_O_2_ and energy. Nat Commun 2025; 16: 1692. 10.1038/s41467-025-57082-4 39956810 PMC11830785

[28] Deng W, Zhu Y, Zhang K et al. Collective electricity generation over the kilovolt level from water droplets. Nano Lett 2025; 25: 7457–64. 10.1021/acs.nanolett.5c01064 40275588

[29] Nie J, Ren Z, Xu L et al. Probing contact-electrification-induced electron and ion transfers at a liquid–solid interface. Adv Mater 2020; 32: 1905696. 10.1002/adma.201905696 31782572

[30] Jin Y, Yang S, Sun M et al. How liquids charge the superhydrophobic surfaces. Nat Commun 2024; 15: 4762. 10.1038/s41467-024-49088-1 38834547 PMC11150272

[31] Ratschow AD, Bauer LS, Bista P et al. How charges separate when surfaces are dewetted. Phys Rev Lett 2024; 132: 224002. 10.1103/PhysRevLett.132.224002 38877904

[32] Lin S, Xu L, Chi Wang A et al. Quantifying electron-transfer in liquid-solid contact electrification and the formation of electric double-layer. Nat Commun 2020; 11: 399. 10.1038/s41467-019-14278-9 31964882 PMC6972942

[33] Wu H, Mendel N, van Der Ham S et al. Charge trapping-based electricity generator (CTEG): an ultrarobust and high efficiency nanogenerator for energy harvesting from water droplets. Adv Mater 2020; 32: 2001699. 10.1002/adma.202001699 32627893

[34] Li Y, Qin X, Feng Y et al. A droplet-based electricity generator incorporating kelvin water dropper with ultrahigh instantaneous power density. Droplet 2024; 3: e91. 10.1002/dro2.91

[35] Wang K, Xu W, Li J et al. Enhancing water droplet-based electricity generator by harnessing multiple-dielectric layers structure. Nano Energy 2023; 111: 108388. 10.1016/j.nanoen.2023.108388

[36] Xu X, Li P, Ding Y et al. Droplet energy harvesting panel. Energy Environ Sci 2022; 15: 2916–26. 10.1039/D2EE00357K

[37] Li Z, Yang D, Zhang Z et al. A droplet-based electricity generator for large-scale raindrop energy harvesting. Nano Energy 2022; 100: 107443. 10.1016/j.nanoen.2022.107443

[38] Gong S, Li K, Sun J et al. Interfacial droplet-based triboelectric nanogenerator with optimized architecture for highly efficient vibrational energy conversion. Joule 2025; 9: 101763. 10.1016/j.joule.2024.09.010

[39] Zhou Y, Zeng Y, Wang J et al. Enhancement of the voltage output of droplet electricity generators using high dielectric high-entropy oxide composites. Energy Environ Sci 2024; 17: 3580–93. 10.1039/D4EE01234H

[40] Wu H, Chen Z, Xu G et al. Fully biodegradable water droplet energy harvester based on leaves of living plants. ACS Appl Mater Interfaces 2020; 12: 56060–7. 10.1021/acsami.0c17601 33264000

[41] Zhang B, Sanjay V, Shi S et al. Impact forces of water drops falling on superhydrophobic surfaces. Phys Rev Lett 2022; 129: 104501. 10.1103/PhysRevLett.129.104501 36112454

[42] Gao X, Jiang L. Water-repellent legs of water striders. Nature 2004; 432: 36. 10.1038/432036a 15525973

[43] Yarin AL . Drop impact dynamics: splashing, spreading, receding, bouncing… Annu Rev Fluid Mech 2006; 38: 159–92. 10.1146/annurev.fluid.38.050304.092144

[44] Vasileiou T, Gerber J, Prautzsch J et al. Superhydrophobicity enhancement through substrate flexibility. Proc Natl Acad Sci USA 2016; 113: 13307–12. 10.1073/pnas.1611631113 27834217 PMC5127354

[45] Choi H, Liang H. Wettability and spontaneous penetration of a water drop into hydrophobic pores. J Colloid Interface Sci 2016; 477: 176–80. 10.1016/j.jcis.2016.05.029 27267040

[46] Song Y, Xu W, Liu Y et al. Achieving ultra-stable and superior electricity generation by integrating transistor-like design with lubricant armor. Innovation 2022; 3: 100301. 10.1016/j.xinn.2022.100301 36051817 PMC9425077

[47] Xu C, Fu X, Li C et al. Raindrop energy-powered autonomous wireless hyetometer based on liquid–solid contact electrification. Microsyst Nanoeng 2022; 8: 30. 10.1038/s41378-022-00362-6 35359613 PMC8918552

