# Supplementary material for: Floating droplet electricity generator on water
Source: Natl Sci Rev. 2025 Aug 4;12(11):nwaf318. doi: 10.1093/nsr/nwaf318 (PMC12530091; doi:10.1093/nsr/nwaf318)
Supplement: nwaf318_Supplemental_Files [file nwaf318_supplemental_files.zip › Supplementary data.pdf]

# Floating droplet electricity generator on water

Wei Deng[1,*], Zihao Wang[1], Jingmin Wang[3], Tao Hu[2], Xiao Wang[2], Xuemei Li[3], Jun Yin[1,2,*] and Wanlin Guo[1,2,*]

[1]State Key Laboratory of Mechanics and Control for Aerospace Structures, Key Laboratory for Intelligent Nano Materials and Devices of the Ministry of Education, Institute for Frontier Science, Nanjing University of Aeronautics and Astronautics, Nanjing 210016, China;

[2]College of Aerospace Engineering, Nanjing University of Aeronautics and Astronautics, Nanjing 210016, China;

[3]College of Materials Science and Engineering, Nanjing University of Aeronautics and Astronautics, Nanjing 210016, China

***Corresponding authors**. E-mails: weideng@nuaa.edu.cn; yinjun@nuaa.edu.cn; wlguo@nuaa.edu.cn

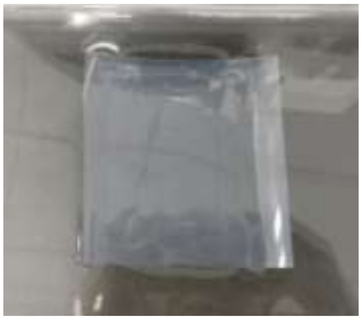

**Figure S1.** A digital photo of a dielectric layer (FEP, 0.2 mm thick) floating on the water surface.

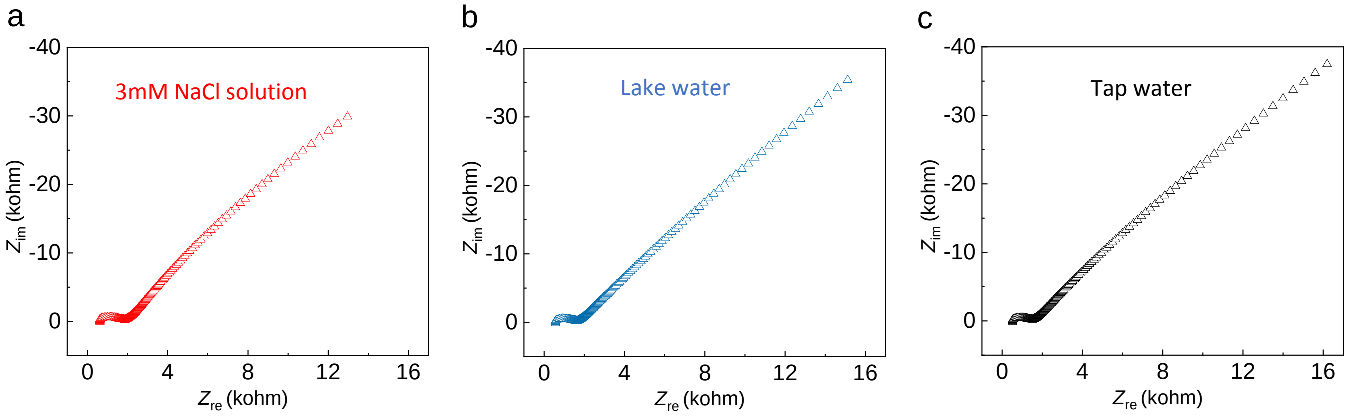


**Figure S2.** Electrochemical impedance spectra of a 3mM NaCl solution, lake water, and tap water. The three solutions have close salinities (0.02%, 0.03%, and 0.02%).

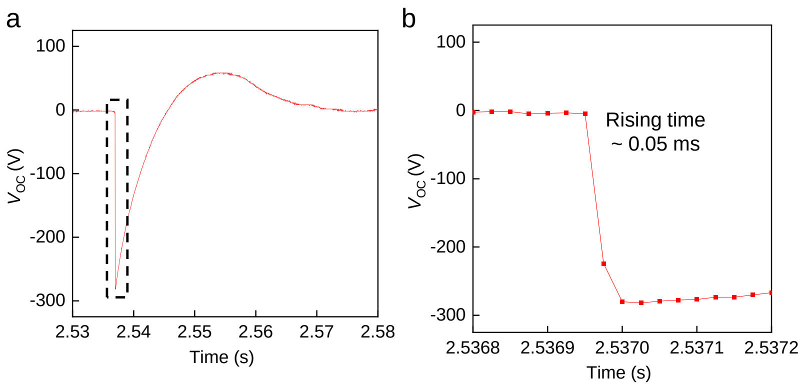


**Figure S3.** An enlarged view of the voltage output pulse of the W-DEG. The rising time of the pulse is about 0.05 ms and the characteristic frequency is approximately 20 kHz.

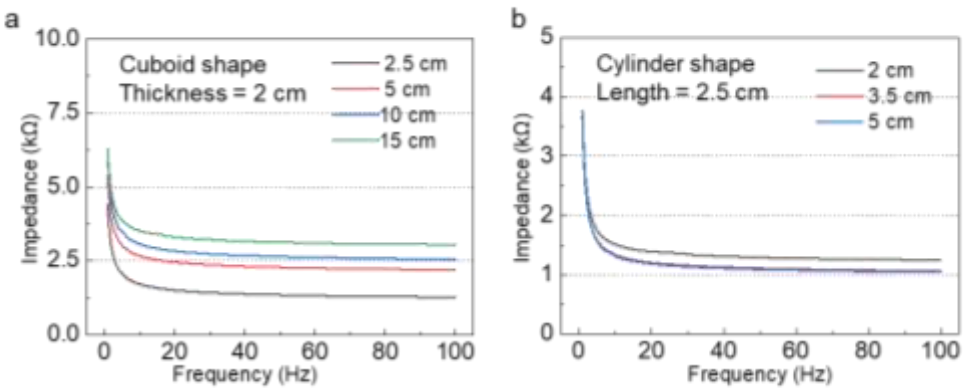


**Figure S4.** Dependence of the water electrode impedances on size and shape. (a) Impedances of the cuboid water electrode of different lengths. (b) Impedances of the cylindric water electrode of different thickness.

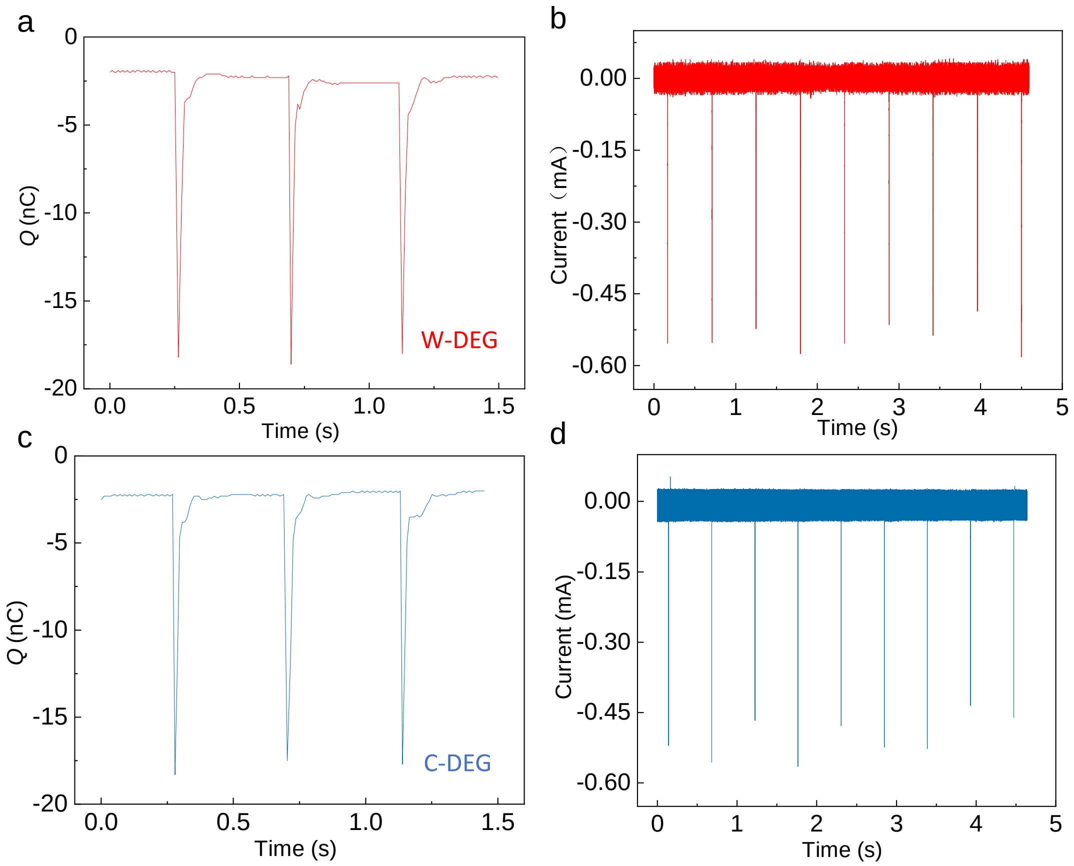


**Figure S5.** Charge transfer and short-circuit current of (a, b) W-DEG and (c, d) C-DEG. The two devices exhibit comparable electrical output performance.

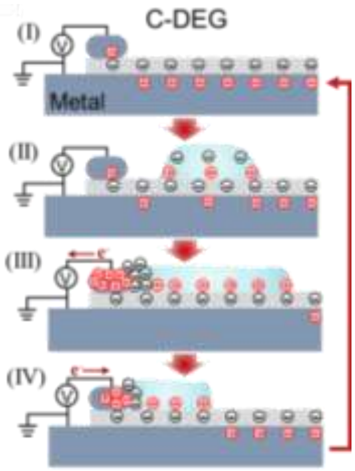


**Figure S6.** Schematic illustration of the charge transfer mechanism in the C-DEG.

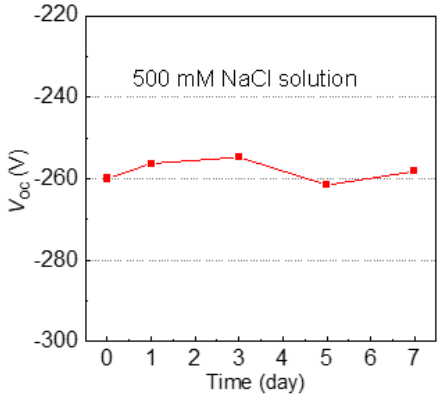


**Figure S7.** Voltage output of the W-DEG floating on a NaCl solution (500 mM) for different days.

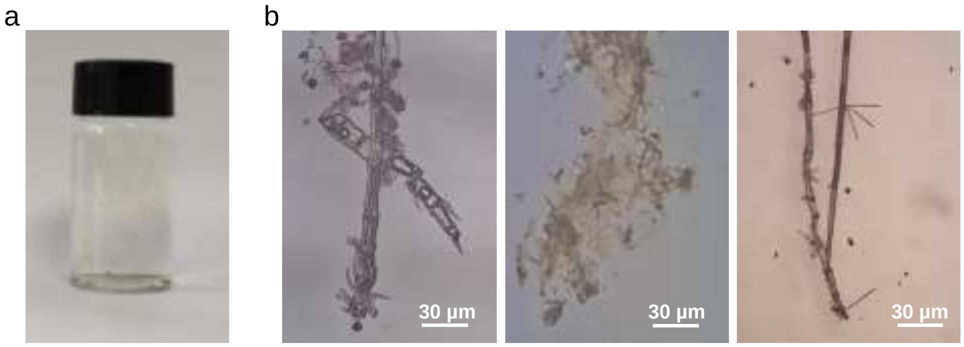


**Figure S8.** Digital photo and microscope images show microorganisms' growth in the collected lake water in the lab after one week.

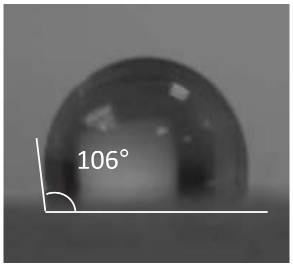


**Figure S9.** Contact angle of water on the FEP film.

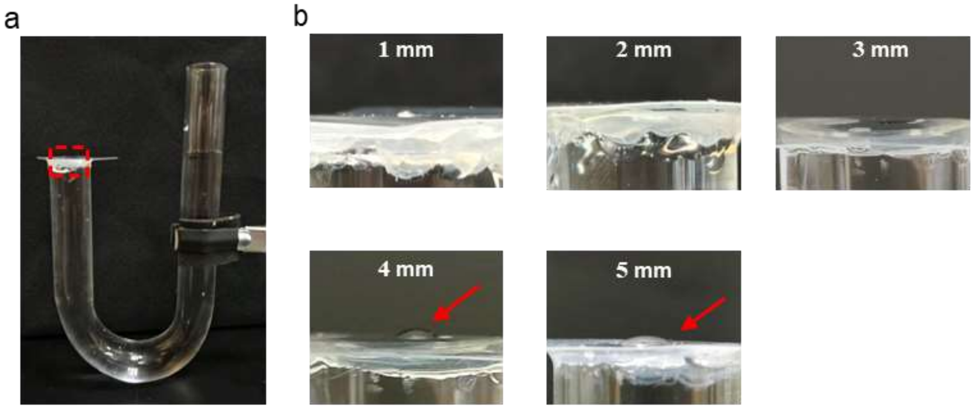


**Figure S10.** Digital photos show (a) the experimental setup and (b) water intrusion in holes of different sizes. Water starts to flow through the hole when the hole size is larger than 3 mm under hydraulic pressure (3 mm $H_2O$).

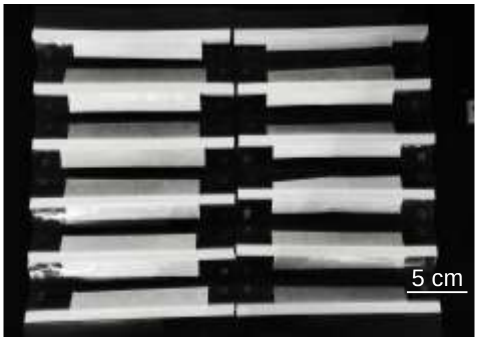


**Figure S11.** Photography of the integrated W-DEG device, consisting of 10 unit devices.

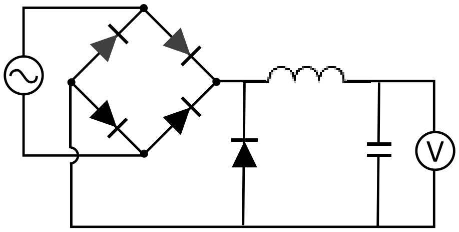


**Figure S12.** Schematic diagram of the power management circuit.
